# Differential effects of synthetic psychoactive cathinones and amphetamine stimulants on the gut microbiome in mice

**DOI:** 10.1371/journal.pone.0227774

**Published:** 2020-01-24

**Authors:** Mariana Angoa-Pérez, Branislava Zagorac, Andrew D. Winters, Jonathan M. Greenberg, Madison Ahmad, Kevin R. Theis, Donald M. Kuhn

**Affiliations:** 1 Research and Development Service, John D. Dingell VA Medical Center, Detroit, Michigan, United States of America; 2 Department of Psychiatry and Behavioral Neurosciences, Wayne State University School of Medicine, Detroit, Michigan, United States of America; 3 Department of Biochemistry, Microbiology and Immunology, Wayne State University School of Medicine, Detroit, Michigan, United States of America; 4 Perinatal Research Initiative in Maternal, Perinatal and Child Health, Wayne State University School of Medicine, Detroit, Michigan, United States of America; University of Illinois, UNITED STATES

## Abstract

The list of pharmacological agents that can modify the gut microbiome or be modified by it continues to grow at a high rate. The greatest amount of attention on drug-gut microbiome interactions has been directed primarily at pharmaceuticals used to treat infection, diabetes, cardiovascular conditions and cancer. By comparison, drugs of abuse and addiction, which can powerfully and chronically worsen human health, have received relatively little attention in this regard. Therefore, the main objective of this study was to characterize how selected synthetic psychoactive cathinones (aka “Bath Salts”) and amphetamine stimulants modify the gut microbiome. Mice were treated with mephedrone (40 mg/kg), methcathinone (80 mg/kg), methamphetamine (5 mg/kg) or 4-methyl-methamphetamine (40 mg/kg), following a binge regimen consisting of 4 injections at 2h intervals. These drugs were selected for study because they are structural analogs that contain a β-keto substituent (methcathinone), a 4-methyl group (4-methyl-methamphetamine), both substituents (mephedrone) or neither (methamphetamine). Mice were sacrificed 1, 2 or 7 days after treatment and DNA from caecum contents was subjected to 16S rRNA sequencing. We found that all drugs caused significant time- and structure-dependent alterations in the diversity and taxonomic structure of the gut microbiome. The two phyla most changed by drug treatments were Firmicutes (methcathinone, 4-methyl-methamphetamine) and Bacteriodetes (methcathinone, 4-methyl-methamphetamine, methamphetamine, mephedrone). Across time, broad microbiome changes from the phylum to genus levels were characteristic of all drugs. The present results signify that these selected psychoactive drugs, which are thought to exert their primary effects within the CNS, can have profound effects on the gut microbiome. They also suggest new avenues of investigation into the possibility that gut-derived signals could modulate drug abuse and addiction via altered communication along the gut-brain axis.

## Introduction

The synthetic psychoactive cathinones (SPCs) are members of a larger class of drugs now referred to as synthetic psychoactive drugs (SPDs). This family also includes synthetic psychoactive cannabinoids, SP opiates and SP hallucinogens. These agents are designed to mimic the actions of known abused drugs and they remain high on the list of the most abused drugs in the USA and across Europe and Asia. Because of their acute and chronic effects, which include cardiovascular, neurological, and infectious disease (i.e., HIV), as well as psychiatric disorders [1–4], and which can progress to liver and kidney failure, rhabdomyolysis and even death [5], these drugs constitute a serious public health crisis [4]. The principal members of the SPC class are mephedrone (4-methyl-methcathinone; Meph), methcathinone (MeCa), methylone and 3,4-methylenedioxypyrovalerone (MDPV). These drugs share remarkable structural similarity with the amphetamine class of psychostimulants, differing only in their possession of a β-keto group. For instance, the β-keto/deketo analogs are cathinone/amphetamine, MeCa/methamphetamine (Meth) and methylone/MDMA. The structural similarity shared by these drugs translates into an extensive overlap in their pharmacological, neurochemical and behavioral effects. The SPCs and amphetamines share the ability to interact with monoamine transporters to cause the release of dopamine (DA), serotonin (5HT) or norepinephrine (NE) [6–8], alter thermoregulation [6,9,10], increase locomotor activity [6,9,11] and serve as discriminative stimuli [9,12,13]. The abuse potential of these drugs has also been affirmed in animal models of addiction [14–16].

While not immediately an obvious target or site of peripheral action for abused drugs, the gut microbiome deserves serious consideration in this regard. The bulk of the human microbiome resides in the GI tract and it has been estimated that the human GI system contains 10^13^−10^14^ microorganisms (same as the number of human cells [17]), which express ~100 times as many genes as the host human genome [18,19]. The gut microbiome is a very dynamic area of research and its normal function is essential to the maintenance of human health. An imbalance in the gut microbiome (i.e., dysbiosis) has also been linked to numerous disease states (e.g., cancer, diabetes [20,21], neurological conditions (e.g., Parkinson’s disease, Alzheimer’s disease; [22]), and psychiatric diseases (e.g., depression and anxiety; [23]). The reciprocal communication between the gut microbiome and the CNS is referred to as the gut-brain axis [24]. With regard to drugs of abuse, a small but growing literature is establishing roles for the gut microbiome in alcohol abuse and withdrawal [25,26], opioid tolerance [27,28], nicotine and smoking [29], cocaine reward [30], and in Meth-induced conditioned place preference [31]. Finally, it has been shown that patients with substance use disorders (SUDs) show changes in gut bacterial diversity [32].

Despite the paucity of published papers in the area of drug abuse and gut microbiome interactions, the premise for undertaking such studies is actually quite compelling for the following reasons: 1) SPCs primarily target the transporters for 5HT (SERT), DA (DAT) and NE (NET) [6,33] to increase extracellular neurotransmitter levels in the brain and these same transporters are highly expressed in the gut [34–36]; 2) these same monoamine transporters are also targets for psychostimulants like Meth and cocaine [8,33]; 3) enhanced monoamine signaling in the gut can change the composition of the microbiome [37] and, in turn, modify gut function [38,39]; 4) production of ammonia from urea is catalyzed by urease enzymes in gut bacteria (the human genome does not encode urease genes) [40], suggesting the microbiome as a site, in addition to the liver, of ammonia production that is known to modulate Meth-induced neurotoxicity; and 5) antibiotics, which deplete the gut microbiome, counteract reinstatement of Meth-seeking behaviors [41,42] and reduce development of an MDPV conditioned place preference [43]. In summary, the gut microbiome is a likely target of the SPCs and the amphetamine-like psychostimulants. Therefore, as a first step in gaining a better understanding of how drugs of abuse might alter gut-brain communication, we have characterized the effects of selected SPCs and amphetamines on the gut microbiome of mice.

## Materials and methods

### Study drugs

(R,S)-N-Methcathinone HCl and (R,S)-mephedrone HCl were provided by the NIDA Research Resources Drug Supply Program. Racemic 4-methylmethamphetamine HCl was synthesized as described by Davis et al. (2012) from methylamine HCl and 4-methylphenylacetone purchased from Alfa Aesar (Ward Hill, MA, USA). (+)- Methamphetamine HCl, was purchased from Sigma-Aldrich (St. Louis, MO, USA). All DEA schedule controlled drugs were purchased under DEA registration numbers RK0237986 (Schedule 1) and RK0245995 (Schedules 2–5).

### Animals and drug treatment

Female C57BL/6 mice (Harlan, Indianapolis, IN, USA) weighing 18–25 g at the time of experimentation were housed 5–7 per cage in large shoe-box cages in a light- (12 h light/dark) and temperature-controlled room. Female mice were used as they have been shown to be impacted by the neurotoxicity induced by amphetamines and to maintain consistency with our previous studies of Meth and β-ketoamphetamine interactions [44–47]. Mice had free access to food and water. The mice used were randomly divided into treatment groups (N = 5–7 mice per group) and were treated through intraperitoneal injection (i.p.) with saline (controls), Meth (5 mg/kg), 4-methylmethamphetamine (4MM; 40 mg/kg), MeCa (80 mg/kg), or Meph (40 mg/kg) in a single-day binge-like regimen, which involves 4 injections (0.2 mL) at 2 h intervals. This binge treatment regimen has been established by multiple prior studies in this laboratory and others to elicit significant neurotoxicity for amphetamine compounds [48–51]. Doses of β-keto amphetamines and 4MM eliciting mild to moderate DA depletion were selected based on prior studies [44–47,52–54]. Mice were sacrificed by decapitation 1, 2 or 7 days after drug treatment and caecum contents were harvested, weighed and stored frozen at -80°C until DNA isolation. Stressors such as noise and handling by multiple persons were avoided and mice were monitored daily for signs of distress or injury until the endpoints at 1, 2 or 7 days. The Institutional Care and Use Committee of Wayne State University approved the animal care and experimental procedures. All procedures were also in compliance with the NIH *Guide for the Care and Use of Laboratory Animals* and were conducted in compliance with ARRIVE guidelines and under IACUC-approved protocols.

### Microbiome analysis

DNA was extracted from caecum contents (~200 mg wet weight) using QIAamp PowerFecal DNA kits and sample DNA concentrations were determined using a Qubit 4 Fluorometer and ranged from 70–100 ng/μl. Samples were sequenced in duplicate on an Illumina MiSeq system using a 2 X 250 cycle V2 kit following Illumina sequencing protocols and with Illumina reagents following the procedures detailed by Kozich and colleagues [55]. The 16S rRNA gene primers used targeted the V4 region of the gene (forward primer: 5’-GTGCCAGCMGCCGCGGTAA-3’; reverse primer: 5’-GGACTACHVGGGTWTCTAAT-3’). The 16S rRNA gene sequences from the paired fastq files were trimmed, screened and aligned using mothur [56], in accordance with the MiSeq SOP established by Schloss and colleagues (https://www.mothur.org/wiki/MiSeq_SOP). After de-multiplexing and quality control (e.g., truncating reads with >2 adjacent low quality base calls; discarding reads containing any ambiguous base calls in surviving sequences), sequences were binned into operational taxonomic units (OTUs) based on percent sequence identity (97%), the OTUs were taxonomically classified in mothur, and the bacterial community data were thereafter visualized and statistically analyzed using PAST software (v3.20; [57]). Microbiome diversity was characterized in terms of α-diversity using the Chao1 (i.e. community richness) and Shannon and Simpson (1-D) (i.e. community heterogeneity) indices. Because the number of sequences per sample was significantly different among treatment groups, subsampling was performed to the level of the least represented sample prior to calculating α-diversity measures. The number of sequences obtained were as follows: 123,742 ± 22,459 (pre-subsampling) with Good’s coverage values of 99.7 ± 0.05 and 42,528 (post-subsampling) with Good’s coverage values of 99.3 ± 0.07. β-diversity was assessed using the Jaccard (i.e. shared composition) and Bray-Curtis (i.e. shared structure) indices based on relative abundance data. High-dimensional class comparisons were carried out with linear discriminant analysis effect size (LEfSe) in an on-line interface [58] using default parameters with the exception that LDA score was set to 3.6. Heat maps were generated using MetaboAnalyst 4.0 [59].

### Data analysis and statistics

The indices for α-diversity were obtained using PAST software (v3.20). The results were analyzed statistically with a one-way ANOVA, and subsequent *post hoc* comparisons were performed with Tukey’s test using GraphPad Prism (v6.07) for Windows (GraphPad Software, La Jolla, CA, USA, www.graphpad.com). The indices for β-diversity were also calculated, and statistical analyses were carried out, using PAST software (v3.20). The results were analyzed using a two-way NPMANOVA, and *post hoc* comparisons were made using one-way NPMANOVAs. Taxonomic distributions at the phylum level (treatment X phylum) and lower taxonomic levels (treatment X time) were analyzed with a two-way ANOVA followed by *post hoc* comparisons using Tukey’s tests in GraphPad Prism.

## Results

### Drug effects at the OTU level

**Fig 1:**
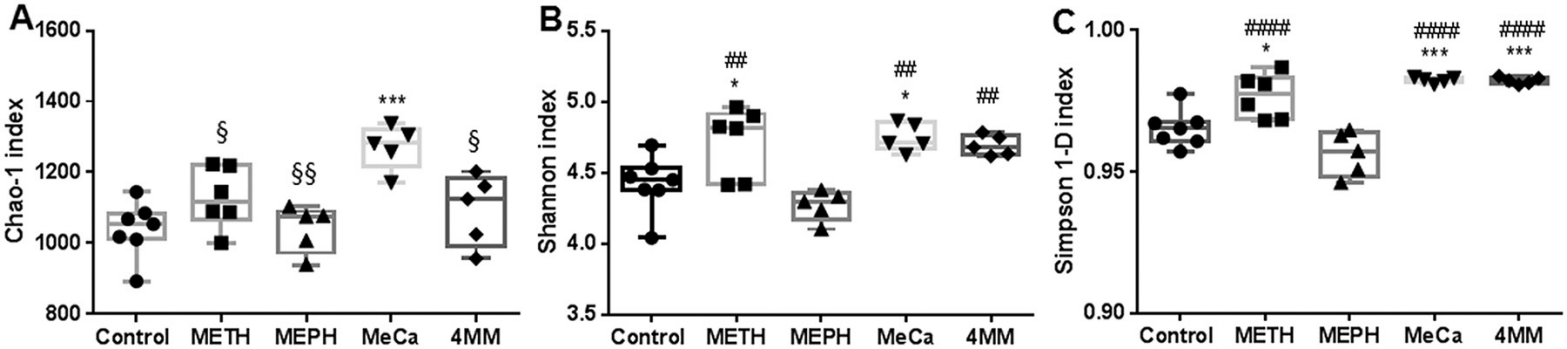
Effects of study drugs on α-diversity. The α-diversity metrics Chao-1 richness estimator (A), Shannon diversity index (B) and Simpson (1-D) index (C) were determined for 16S rRNA gene profiles of caecum contents harvested 1 day after treatment. The individual values for all subjects in each treatment group are included in each box plot. *, p < 0.05 or *** p < 0.001 compared to control; # p < 0.05, ## p < 0.01 or #### p < 0.0001 compared to Meph; § p < 0.05, or §§ p < 0.01 compared to MeCa.

Study drugs caused drug- and time-dependent alterations in the richness and heterogeneity of the gut microbiome at the OTU level as shown in Fig 1. α-diversity values were based on 3 metrics (Chao-1 richness estimator, Shannon diversity index and the Simpson (1-D) index) for 16S rRNA gene profiles for each study drug. The effects of drug treatments on all α-diversity metrics were tested statistically with a one-way ANOVA (F_4,23_ = 7.52, p = 0.0005 for Chao-1; F_4,23_ = 8.91, p = 0.0002 for Shannon; and F_4,23_ = 19.41, p = 0.0001 for Simpson (1-D)) followed by *post hoc* Tukey’s tests. At 1 day after treatment, MeCa was the only treatment in which the gut microbiome exhibited increased richness in comparison to controls (p < 0.001). The microbiomes of MeCa-treated mice were also more OTU-rich than those of mice receiving Meth (p < 0.05), Meph (p < 0.01) and 4MM (p < 0.05) (Fig 1A). The pattern of drug effects on gut microbiome heterogeneity at the 1-day time point, using the Shannon index, was similar to that seen for richness in that Meth and MeCa significantly increased heterogeneity in comparison to controls (p < 0.05 for both), and all drugs significantly increased microbiome diversity in comparison to Meph (Fig 1B; p < 0.05 for all). The Simpson (1-D) index shown in Fig 1C also revealed that Meth, MeCa and 4MM exhibited increased microbiome heterogeneity compared to controls (Meth p < 0.05; MeCa & 4MM: p < 0.001 for both) and Meph (p < 0.0001 for all). The microbiomes of Meph-treated mice did not differ from those of controls using any α-diversity metric. The alterations in taxonomic richness and heterogeneity caused by these drugs at 1 day were not present at either 2 or 7 days post-treatment (S1 Fig).

**Fig 2:**
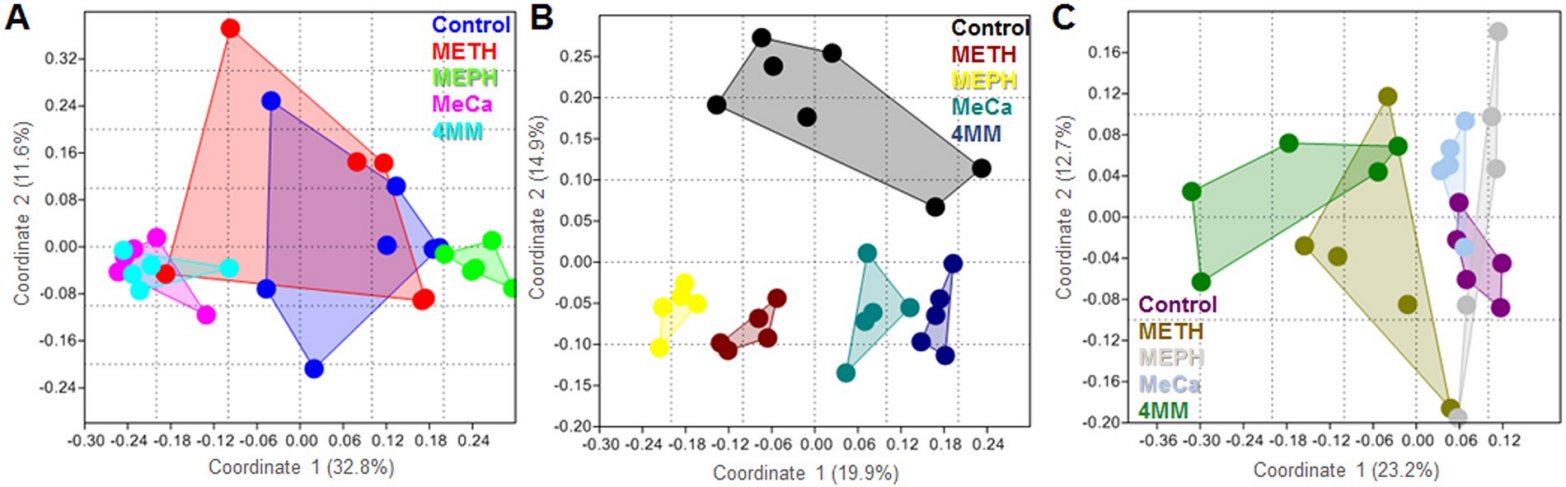
Effects of study drugs on β-diversity. Principal Coordinates Analyses (PCoA) illustrating differences in the structure (i.e. Bray-Curtis index) of gut microbiome profiles among mice treated with the different study drugs. Profiles were generated at 1 (A), 2 (B) or 7 days (C) after drug treatments.

The effects of the study drugs on gut microbiome β-diversity at the OTU level were first tested statistically using a two-way NPMANOVA and the results revealed highly significant main effects for drug (F_4,65_ = 3.35; p = 0.0001) and time (F_2,65_ = 8.43, p = 0.0001), as well as a significant interaction (F_8,65_ = 1.77, p = 0.0001). It can be seen in Fig 2 that there was separation in gut microbiome structure (i.e., Bray-Curtis index) among mice treated with different study drugs. While some overlapping of treatment groups on a two dimensional PCoA plot was visible at 1 day post drug injections, all pairwise comparisons among controls and drug treatments were significantly different (Fig 2A; p < 0.02, NPMANOVA). Fig 2B shows the effects of drugs on gut microbiome structure 2 days after treatment, and the microbiomes of mice from all treatments clustered well apart from each other. All pairwise comparisons among controls and drugs were highly significant (p < 0.016; NPMANOVA). Finally, Fig 2C shows that the gut microbiome profiles at 7 days clustered apart for all treatments, and all pairwise comparisons were again significant (p < 0.029; NPMANOVA), except for the comparison of Meph to MeCa (p = 0.062). The results of all statistical tests of microbiome structure are included in S1 Table. The Jaccard Similarity Index was also used to test for variation in gut microbiome composition and the results were similar to those observed for the Bray-Curtis Index comparisons (S2 Table). There was a high degree of separation among the bacterial profiles seen for controls and all drugs at 1 and 2 days after treatment, as shown in S2 Fig. All pairwise comparisons among treatment conditions were significant at both time points (p < 0.04; NPMANOVA), except for the comparison of MeCa to 4MM at the 1-day time point (p = 0.055; S2 Table). By 7 days post-treatment, the bacterial profiles were not as clearly separated (S2 Fig), which was reflected by far fewer significant differences among the treatment groups (S2 Table).

**Fig 3:**
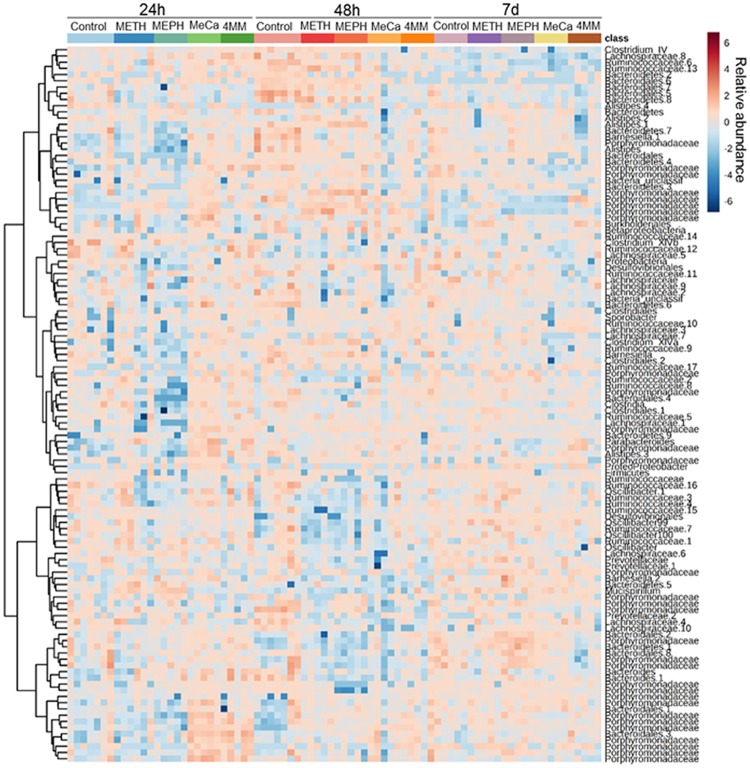
Heat map illustrating the relative abundances of OTUs after treatment with study drugs. The most prominent OTUs (≥ 1% average relative abundance) among treatment groups are plotted for each drug at 1, 2 h or 7 days after drug injections. Clustering was done using the Ward algorithm.

The taxonomic identities of prominent OTUs (i.e., ≥ 1% relative abundance among all subjects considered collectively) for each drug revealed broad variance in drug- and time-dependent effects. These results are presented in the heat map in Fig 3 and include results from all subjects. A large number of OTUs were decreased 1 day after treatment with Meth, Meph and MeCa. These OTUs were largely classified as Porphyromonadaceae, Bacteroidales, Clostridiales and Ruminococcaceae (Fig 3). Decreases in these same taxa were evident after treatment with 4MM, but the changes were smaller in magnitude than was seen after treatment with Meth, Meph and MeCa. By 2 days post-treatment, decreases were less numerous and several increases in taxa were seen after injections of Meth (Lachnospiraceae and Bacteroidetes) and Meph (Ruminococcaceae and Porphyromonadaceae). On the other hand, mice treated with MeCa still exhibited broad decreases in bacterial taxa, including Bacteroidetes, Ruminococcaceae and Lachnospiraceae. At the 7 day time point, all drugs caused decreases in several taxa, especially *Clostridium IV*, Ruminococcaceae, *Alistipes*, Porphyromonadaceae and *Oscillibacter*. Moderate increases in *Barnesiella* and Bacteroidales were however seen 7 days after treatment with Meth and Meph.

**Fig 4:**
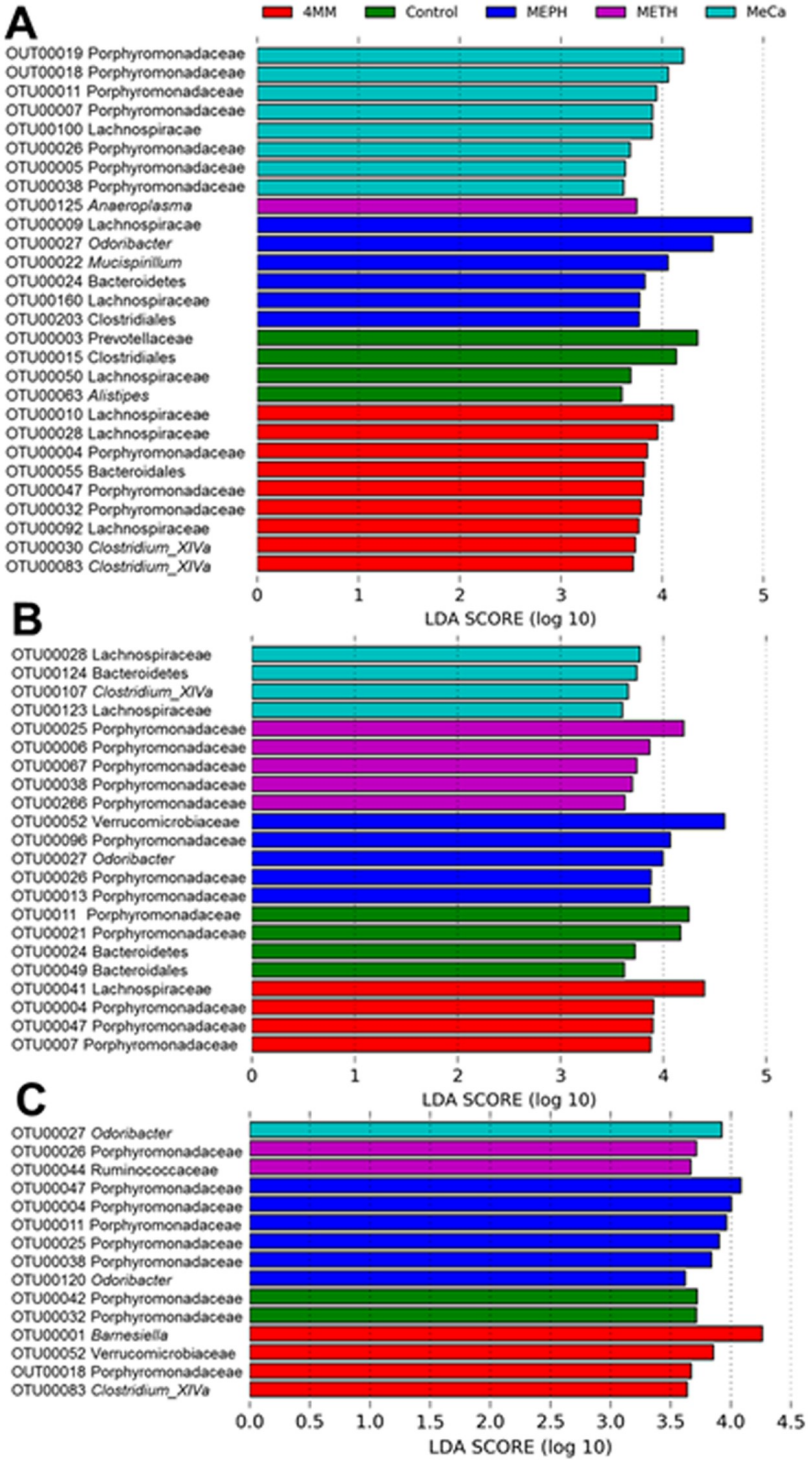
Bacterial taxa that were differentially abundant across study drug treatments. Linear discriminant analysis effect size (LEfSe) was carried out and the results are presented for taxa with LDA scores of > 3.6 for the treatment groups at 1 (A), 2 (B) and 7 days (C) after treatments.

Fig 4 presents results from linear discriminant analysis effect size (LEfSe) analysis and highlights the effect size of the study drug treatments on affected taxa. It can be seen in Fig 4A that the relatively more abundant OTUs varied for each treatment group compared to the other groups at the 1-day time point. For instance, the discriminant taxa for MeCa were Porphyromonadaceae and Lachnospiraceae, whereas the taxon with relatively more abundance after Meth treatment was *Anaeroplasma*. Meph treatment was marked by *Odoribacter*, *Musispirillum* and Clostridiales, whereas 4MM was marked by abundant OTUs classified as *Clostridium XIVa*, in addition to Lachnospiraceae, Porphyromonadaceae and Bacteroidales. OTUs classified as Prevotellaceae, Clostridiales, Lachnospiraceae and *Alistipes* were relatively more abundant in the control group at the 1-day time point. The OTUs that were relatively abundant at the 2-day time point (Fig 4B) were quite different for each drug by comparison to the 1-day time point. For instance, 7 of 8 OTUs that were differentially abundant 1 day after MeCa treatment were from the family Porphyromonadaceae, but none of these OTUs characterized MeCa at the 2-day time point. Porphyromonadaceae (3 of 4 OTUs) and Lachnospiraceae (1 of 4 OTUs) remained differentially abundant 2 days after treatment with 4MM, in line with their relative abundance at 1 day post-treatment (6 of 9 OTUs). In general, the number of OTUs that were identified as differentially abundant for the study drugs decreased with the passage of time since drug injections, especially for MeCa and 4MM. Notably, Meth was the study drug with the fewest number of discriminating OTUs. The effects of Meph treatment were relatively constant over time with regard to the number of OTUs (5–6) that were relatively more abundant in this treatment than in others; by the 7-day time point (Fig 4C), 5 of 6 OTUs demarking Meph were from the family Porphyromonadaceae.

### Drug effects at the phylotype level

**Fig 5:**
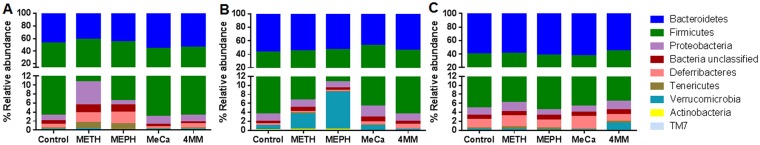
Relative abundances of phyla after treatment with study drugs. Results are presented as % relative abundance of each phylum for each study drug. Stacked columns for the 7 most prominent phyla are included for the 1- (A), 2- (B) or 7- day (C) time points after drug injections.

In light of the changes seen at the OTU level, it was important to next evaluate the effects of the study drugs on specific bacterial phyla. Therefore, drug effects on the percent relative abundance of represented bacterial phyla were quantified and the results are presented in Fig 5. For these analyses, the factor of time was considered as an independent variable. As expected, the greatest number of changes occurred in the Firmicutes and Bacteroidetes phyla. For the 1-day time point (Fig 5A), the main effect of drug was not significant but the main effect of phylum (F_8,207_ = 657.9, p = 0.0001), and the interaction of drug X phylum (F_32,207_ = 1.97, p = 0.0025), was significant. *Post hoc* comparisons revealed that MeCa (p < 0.01) and 4MM (p < 0.01) caused significant increases in percent relative abundance of Firmicutes versus controls. No other pairwise comparisons among the control group and drugs were significant with regard to Firmicutes. As seen above for *Firmicutes*, MeCa (p < 0.01) and 4MM (p < 0.05) also significantly increased the percent relative abundance of Bacteroidetes. Mice treated with Meth (p < 0.001) and Meph (p < 0.05) were significantly lower than those treated with MeCa and 4MM for the percent relative abundance of Bacteroidetes. Fig 5B shows that Meph significantly changed the percent relative abundance of Verrucomicrobia at the 2-day time point by comparison to controls (p < 0.01), MeCa and 4MM (p < 0.05 for both). MeCa was also significantly different from control (p < 0.01), Meth and Meph (p < 0.01 for both) with regard to percent relative abundance of Firmicutes, whereas changes in *Bactoidetes* were restricted to MeCa versus controls (p < 0.001), Meth and 4MM (p < 0.05 for both). None of the other pairwise comparisons were altered significantly at the 2-day time point. Several changes in percent relative abundance persisted for 7 days after drug treatments as seen in Fig 5C. Specifically, 4MM was significantly reduced by comparison to MeCa for Firmicutes, as well as in comparison to controls (p < 0.001). For Bacteroidetes, 4MM was different from control (p < 0.05) and from Meph and MeCa (p < 0.001 for both).

**Fig 6:**
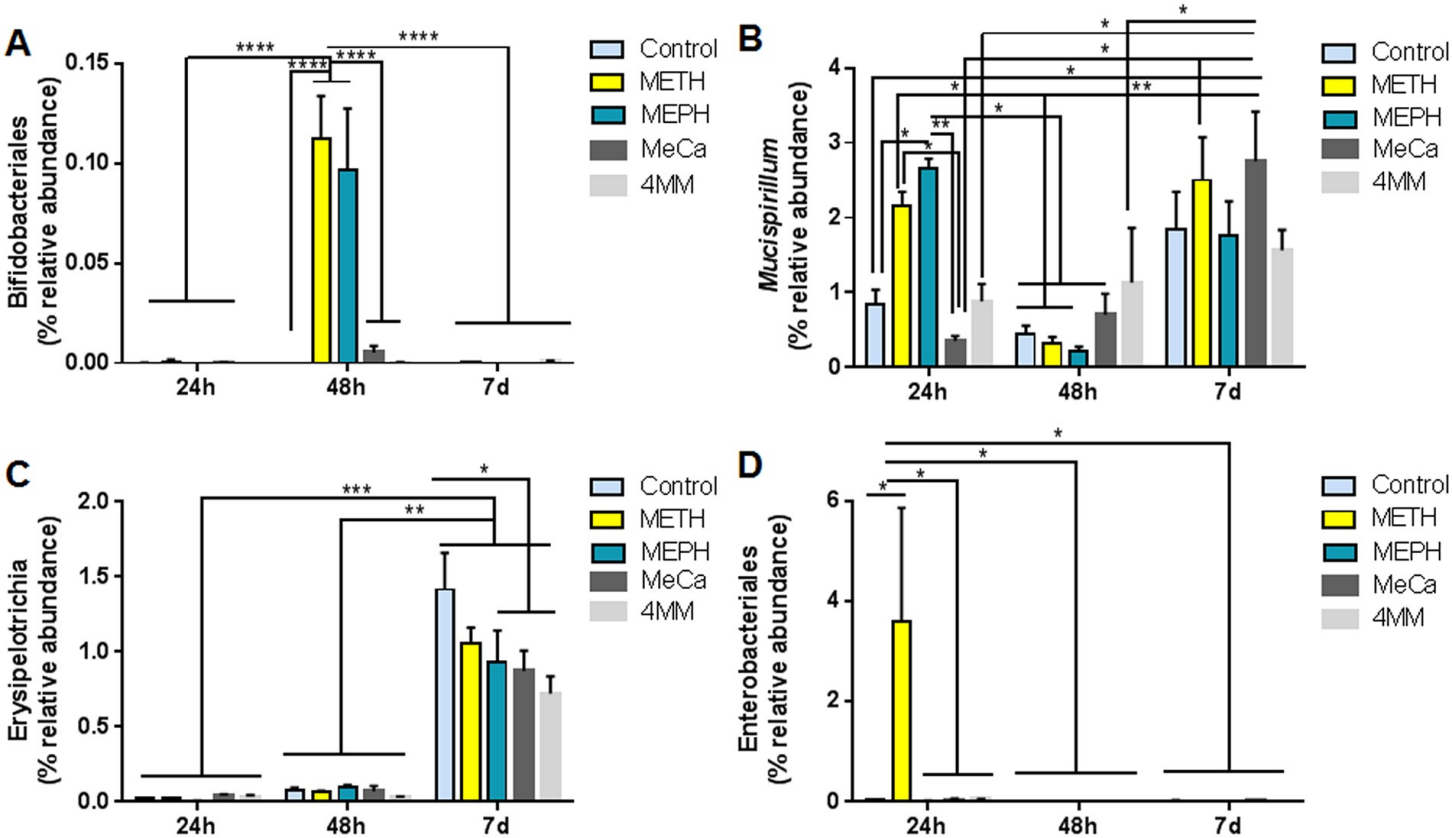
Effects of study drugs on selected taxa below the level of phylum. Results are presented as % relative abundance of taxa 1, 2 or 7 days after drug injections for Bifidobacteriales (A), *Mucispirillum* (B), Erysipelotrichia (C) and Enterobacteriales (D). * p < 0.05, ** p < 0.01, *** p < 0.001 and **** p < 0.0001 for the comparisons demarked by connecting lines above the bars.

Specific drug effects on taxa below the level of phylum were also probed in view of the fact that changes at the highest taxonomic level may not have reached statistical significance because of increases and decreases of equal magnitude in percent relative abundance at lower taxonomic levels. Fig 6 shows these results and indicates that effects at the taxonomic levels of class, order or genus varied according to the study drug in a time-dependent manner. A few illustrative examples are presented in Fig 6 and remaining comparisons are included as supplemental figures (S3 Fig). Fig 6A shows that Meth and Meph significantly increased the percent relative abundance of Bifidobacteriales at the 2-day time point in comparison to all other drugs and times. Fig 6B shows the complex interaction of both drug and time in influencing the percent relative abundance of *Mucispirillum*, which was increased significantly at the 1- and 7- day time points and yet was relatively unchanged 2 days after treatments. In contrast to these time-lines, Fig 6C shows that all treatment conditions significantly increased the percent relative abundance of Erysipelotrichia at the 7-day time point. Finally, Fig 6D shows that Meth was the only treatment to significantly increase the percent relative abundance of Enterobacteriales, and that this effect was restricted to the 1-day time point. A number of additional drug- and time-dependent effects on the relative abundances of various taxa are included in S3 Fig. In addition, the results of all statistical analyses for the data in Fig 6 and S3 Fig are included in S3 Table.

A BLAST analysis (i.e., comparison of 16S rRNA representative gene sequences to those in the BLAST taxonomy database) for all OTUs revealed as differentially abundant in the LEfSe analysis (Fig 4) identified (i.e. > 99.6% identity) several bacterial species, including *Fusimonas intestini* and *Mucispirillium intestinale* for Meph, *Duncaniella muris* and *Paramuribaculum intestinale* for MeCa, and *Muribaculum intestinale* for 4MM at the 1-day time point. Meph was signified by *Akkermansia muciniphila* and *Paramuribaculum intestinale* at the 2- and 7-day time points, respectively. These results are summarized in Table 1.

**Table 1:** BLAST analysis identifying individual bacterial species linked to specific study drugs.

1 d			
OTU	Bacterial species	Study drug	% sequence identity
OTU0009	*Fusimonas intestini*	Meph	99.6
OTU00022	*Mucispirillum schaedleri*	Meph	100
OTU00018	*Duncaniella muris*	MeCa	100
OTU00011	*Paramuribaculum intestinale*	MeCa	100
OTU00032	*Muribaculum intestinale*	4MM	100
**2 d**			
OTU00011	*Paramuribaculum intestinale*	Control	100
OTU00052	*Akkermansia muciniphila*	Meph	100
**7 d**			
OTU00018	*Duncaniella muris*	4MM	100
OTU00011	*Paramuribaculum intestinale*	Meph	100

The consensus sequence for the OTUs identified as being differentially representative for each study drug in the LEfSe analysis was obtained in mothur and queried against the BLAST taxonomy database. Only those sequences with > 99% identity to a characterized bacterial species are included in the table.

## Discussion

The goal of the present study was to determine if selected drugs of abuse alter the gut microbiome. The results were clear in showing that Meth, Meph, MeCa and 4MM each caused significant alterations in the diversity and taxonomic structure of the gut microbiome of mice. Three different metrics for assessing α-diversity established that all study drugs caused significant changes in microbiome richness and heterogeneity. Specifically, Meth, MeCa and 4MM increased microbiome α-diversity, whereas Meph generally resulted in a small but significant decrease. These changes occurred within 1 day of drug treatment and microbiome α-diversity returned to control levels by 2 and 7 days post-treatment. With regard to β-diversity, all drugs caused significant alterations in microbiome composition and structure that were apparent within 1 day and which persisted for 2 to 7 days after treatment. These changes in the gut microbiome caused by Meth, Meph, MeCa and 4MM are notable for the rapidity of their onset as well as for their persistence.

All study drugs also caused significant alterations in the taxonomic makeup of the gut microbiome. By and large, the predominant changes in OTU structure occurred within the Firmicutes and Bacteroidetes phyla. This pattern was expected in light of the fact that the mouse microbiome is dominated by these two phyla [60]. Individual subject responses to the study drugs over time, as shown in the heat map, revealed widespread alterations in the relative abundances of individual OTUs that were time-dependent. These changes occurred widely throughout the Firmicutes and Bacteroidetes phylogenetic trees and could be traced to the levels of order and genus, and in some cases to specific species. For instance, Meth, Meph and MeCa caused decreases predominantly in Porphyromonadaceae, Bacteroidales, Clostridiales and Ruminococcaceae. Increases seen after these same drugs were dominated by Lachnospiraceae and Ruminococcaceae. The changes that occurred below the phylum level revealed the complex nature by which the study drugs altered the gut microbiome. In most cases, the effects were drug- and time-specific. For instance, only Meth and Meph increased the relative abundance of Bifidobacteriales and these effects occurred at the 2-day time point only. All study drugs increased *Mucispirillum* abundance at 1 day post-treatment and these changes nearly returned to control levels at the 2-day time point and then increased once again by 7 days post-treatment. An increase in the relative abundance of Enterobacteriales was selective for Meth and only occurred at the 1-day time point. Finally, all study drugs significantly increased the relative abundance of Erysipelotrichia but this effect did not emerge until 7 days after treatments.

LEfSe analysis identified numerous bacterial taxa that were relatively more abundant in each treatment group by comparison to the other groups and, once again, these taxonomic “biomarkers” showed considerable variation among drugs and times. MeCa and 4MM were dominated by Porphyromonadaceae and Lachnospiraceae at the 1-day time point and by 2 days post-treatment, Porphyromonadaceae was no longer discriminant for MeCa but remained so for 4MM. Meph had perhaps the broadest effects on microbiome structure and significantly enriched 7 different taxa at the 1- and 2-day time points. These taxa were Lachnospiraceae, *Odoribacter*, *Mucispirillum*, Bacteroidales, Clostridiales, Verrucomicrobiaceae and Porphyromonadaceae. By contrast, Meth was associated with the fewest numbers of differentially abundant taxa: *Anaeroplasma* (at 1 day), Porphyromonadaceae (at 2 days) and Ruminococcaceae (7 day). In general, the number of discriminant taxa for all study drugs diminished over time, reaching the fewest number by 7 days post-treatment. Studies with other psychostimulants affecting the dopaminergic system include reports of chronic cocaine treatment leading to similar beta diversity alterations, and enrichments of members of Lachnospiracaceae and Ruminococcaceae [61].

The changes in the gut microbiome caused by Meth, Meph, MeCa and 4MM were so drug- and time-specific, it is difficult to discern a defining pattern that can be linked to a functional aspect of these drugs of abuse. This is very surprising and unexpected in light of the structural, behavioral and neurochemical features shared by these drugs. Perhaps the one property that most distinguishes these drugs is neurotoxicity. In this regard, studies suggest that minor alterations in the phenethylamine structure of these drugs are important determinants of neurotoxic potential, and that the addition of either a β-keto or 4-methyl substituent to Meth (i.e., to result in MeCa or 4MM, respectively) significantly diminishes neurotoxicity, whereas the addition of both to Meth (i.e., to result in Meph) obviates neurotoxicity [47]. While limited in number, some studies support the possibility that the gut microbiome can at least modulate Meth-induced neurotoxicity. Mythramycin, used as a transcriptional inhibitor [62], and ceftriazone, used to increase expression of the glutamate transporter [63], are also antibiotics and each protects against Meth damage to the dopamine neuronal system. Minocycline is also neuroprotective against Meth [64].

Recent studies with drugs that cause Parkinson’s disease-like damage to the dopamine neuronal system, reported that they also induce gut dysbiosis [65,66]. This microbial imbalance was evidenced by increases in Enterobacteriaceae, and particularly of *Proteus mirabilis*, a species within this family [67]. Interestingly, a caecal enrichment of *P*. *mirabillis* was also described after treatment with MDMA [68]. One of the known effects of synthetic cathinones and amphetamines is their ability to induce hyperthermia [47]. In this regard, recent studies have revealed that not only gut microbiota can impact thermoregulatory processes [69], but also that the hyperthermic effects of amphetamine drugs are attenuated by antibiotics [68]. Furthermore, *P*. *mirabilis* is characterized by its high levels of urease [70], which hydrolyses urea to ammonia. Increased production of ammonia by Meth has been linked to its ability to cause neurotoxicity to dopamine nerve endings [71,72]. We noted that Meth increased the relative abundance of Proteobacteria by comparison to Meph (Fig 5) and significantly increased the abundance of Enterobacteriales (Fig 6D), the taxonomic order above *P*. *mirabilis*. These changes were not observed after treatment of mice with the non-neurotoxic Meph. This is a preliminary and speculative association between Meth-induced neurotoxicity and alterations in the gut microbiome that suggests new avenues of investigation.

The link between the study drugs used presently and the gut microbiome is strengthened by three additional factors. First, at least Meth can alter GI function in humans by causing intestinal ischemia [73] and infarction [74], and it can also lead to reductions in GI motility and paralytic ileus [75]. In animals, self-administration of Meth increases colon permeability [76] and gut toxicity [77]. Second, psychostimulants have long been used to suppress appetite as an aid to weight loss [78] and it is known that individuals with eating disorders have worsened symptoms and poorer outcomes if they co-abuse stimulants such as Meth [79]. Third, there is increasing evidence of the capacity of the gut microbiome to modify the pharmacokinetics and metabolism of drugs [80]. Early studies have described that Meth can be demethylated by intestinal bacteria into amphetamine, norephedrine and an unknown compound [81]. This biotransformation of Meth can be achieved by *Lactobacilli*, *Enterococci* and *Clostridia*, and would likely result in decreased drug activity [80]. These microbial actions could contribute to the differential toxicity of Meth compared to Meph, MeCa and 4MM but further studies are needed to confirm this notion. Therefore, drugs of abuse that alter appetite and GI function may do so via interactions with the gut microbiome.

This current study has several strengths. First, it is the first characterization of the effects of several important drugs of abuse on the gut microbiome. Drugs of abuse have not been studied extensively with regard to their ability to cause gut dysbiosis, probably because it is generally held that these drugs exert their addictive effects entirely within the CNS. The present results therefore serve as an impetus for expanded searches for gut-derived substances that could mediate substance abuse and addiction. Second, we show that all study drugs cause changes in gut microbiome richness and structure that are rapid in onset and that persist for at least 7 days. Third, our results demonstrate the complex interaction between individual drugs and time in modifying the gut microbiome. Despite the remarkable similarities in structure shared by our study drugs, their effects on the gut microbiome were highly distinct.

This study has several limitations. First, this was a molecular microbiology study without experiments designed to link drug-induced alterations in the microbiome to functional changes in physiology and behavior. Second, we treated mice with a binge-like regimen that was completed in a single day whereas humans with SUDs self-administer psychostimulants (e.g., methamphetamines and cocaine) in a binge pattern that repeats at intervals throughout a day and continues for many succeeding days or months [82,83]. Third, we tested single drugs for their effects on the gut microbiome and human drug abusers generally abuse several drugs simultaneously. For instance, Meth addicts frequently co-abuse alcohol [83] and SPCs are commonly taken with other drugs of abuse to include alcohol, Ecstasy and cannabis [84]. Additional studies are therefore needed to assess poly-drug effects on the gut microbiome. Fourth, animals were group-housed in single cages per treatment so nesting effects were not discerned.

In conclusion, the present study establishes that important psychoactive drugs of abuse cause significant alterations in the gut microbiome. Despite sharing remarkable structural similarities, the study drugs caused distinct changes in the gut microbiome that were rapid in onset and relatively long-lived. Our results establish an initial foundation upon which future studies can build by investigating longer-term exposure to individual drugs and to drug combinations favored by individuals with SUDs. It is not yet possible to relate the present results in mice to humans with SUDs, given that the only report of the effects of Meth on the gut microbiome in humans combined Meth, heroin, ephedrine, alcohol and tobacco into one group, so it is not possible to discern drug-specific effects [32]. Finally, as more research strengthens a link between drugs of abuse (and addiction) and the gut microbiome, additional studies can use treatments that target the microbiome (e.g., antibiotics, microbiome transplantation, probiotics) to reduce substance abuse and relapse.

## Supporting information

S1 Fig Effects of study drugs on α-diversity. The α-diversity metrics Chao-1 richness estimator (A,D), Shannon diversity index (B,E) and Simpson (1-D) index (C,F) were determined for 16S rRNA gene profiles of caecum contents harvested 2 (A-C) or 7 days (D-F) after treatment. The individual values for all subjects in each treatment group are included in each box plot. None of the treatments were statistically different from controls at either time point.
(TIF)

S2 Fig Effects of study drugs on β-diversity. Principal Coordinates Analyses (PCoA) illustrating differences in 16S rRNA gene profiles among the study drugs. Profiles were generated for 16S rRNA gene community structure using the Jaccard index at 1 (A), 2 (B) or 7 days (C) after drug treatments.
(TIF)

S3 Fig Effects of study drugs on selected taxa below the level of phylum. Results are presented as % relative abundance of taxa 1, 2 or 7 days after drug injections for Bacteroidia (A), Coriobacteriales (B), Betaproteobacteria (C), Burkholderiales (D), Clostridia (E), Verrucomicrobiae (F), Desulfovibrionales (G), Mollicutes (H) and Pasteurellales (I). * p < 0.05, ** p < 0.01, *** p < 0.001 and **** p < 0.0001 for the comparisons demarked by connecting lines above the bars.
(TIF)

S1 Table NPMANOVA statistical test results for Bray-Curtis pairwise comparisons. Cell entries are p values for the indicated statistical comparisons among controls and drug treatments.
(DOCX)

S2 Table NPMANOVA statistical test results for Jaccard pairwise comparisons. Cell entries are p values for the indicated statistical comparisons among controls and drug treatments.
(DOCX)

S3 Table Statistical comparisons for data in Fig 5 and S3 Fig. Cell entries are p values for the indicated statistical comparisons among controls and drug treatments. The symbols are * p < 0.05; ** p < 0.01; *** p < 0.001; **** p < 0.0001.
(XLSX)
