# Supplementary figures and images for: Differential effects of synthetic psychoactive cathinones and amphetamine stimulants on the gut microbiome in mice
Source: PLoS One. 2020 Jan 24;15(1):e0227774. doi: 10.1371/journal.pone.0227774 (PMC6980639; doi:10.1371/journal.pone.0227774)

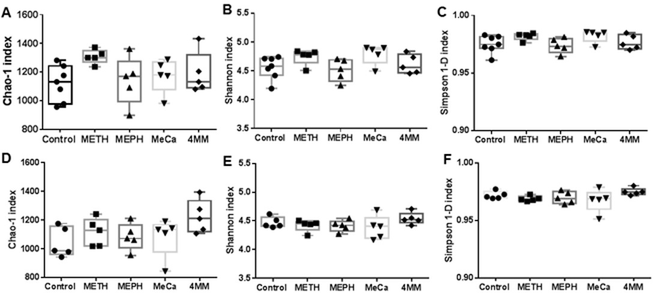

Supplement: S1 Fig — The α-diversity metrics Chao-1 richness estimator (A,D), Shannon diversity index (B,E) and Simpson (1-D) index (C,F) were determined for 16S rRNA gene profiles of caecum contents harvested 2 (A-C) or 7 days (D-F) after treatment. The individual values for all subjects in each treatment group are included in each box plot. None of the treatments were statistically different from controls at either time point. (TIF) [file pone.0227774.s001.tif]

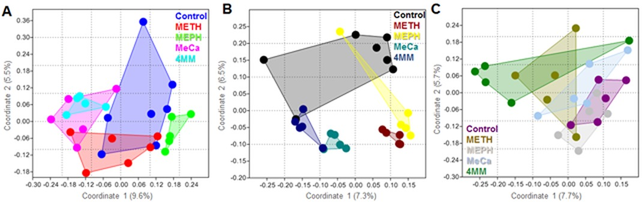

Supplement: S2 Fig — Principal Coordinates Analyses (PCoA) illustrating differences in 16S rRNA gene profiles among the study drugs. Profiles were generated for 16S rRNA gene community structure using the Jaccard index at 1 (A), 2 (B) or 7 days (C) after drug treatments. (TIF) [file pone.0227774.s002.tif]

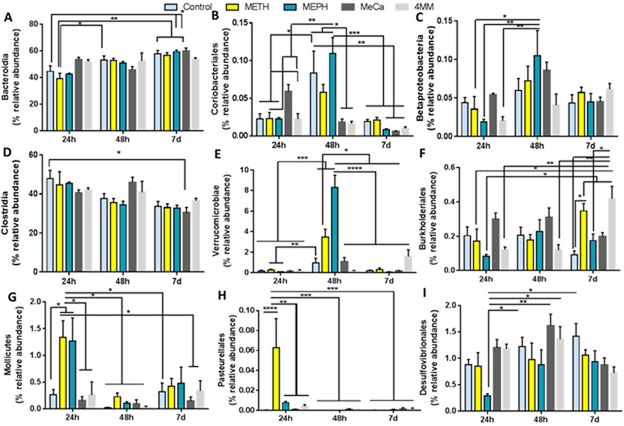

Supplement: S3 Fig — Results are presented as % relative abundance of taxa 1, 2 or 7 days after drug injections for Bacteroidia (A), Coriobacteriales (B), Betaproteobacteria (C), Burkholderiales (D), Clostridia (E), Verrucomicrobiae (F), Desulfovibrionales (G), Mollicutes (H) and Pasteurellales (I). * p < 0.05, ** p < 0.01, *** p < 0.001 and **** p < 0.0001 for the comparisons demarked by connecting lines above the bars. (TIF) [file pone.0227774.s003.tif]
