# Supplementary material for: Differential effects of synthetic psychoactive cathinones and amphetamine stimulants on the gut microbiome in mice
Source: PLoS One. 2020 Jan 24;15(1):e0227774. doi: 10.1371/journal.pone.0227774 (PMC6980639; doi:10.1371/journal.pone.0227774)
Supplement: S1 Table — Cell entries are p values for the indicated statistical comparisons among controls and drug treatments. (DOCX) [file pone.0227774.s004.docx]

| **S1 Table. NPMANOVA statistical test results for Bray-Curtis pairwise comparisons** | | | | | |
| --- | --- | --- | --- | --- | --- |
| 24h | | | | | |
|  | Control | Meth | Meph | MeCa | 4MM |
| Control |  | 0.027 | 0.001 | 0.001 | 0.0013 |
| Meth | 0.027 |  | 0.0034 | 0.0056 | 0.0086 |
| Meph | 0.001 | 0.0034 |  | 0.0083 | 0.0095 |
| MeCa | 0.001 | 0.0056 | 0.0083 |  | 0.0064 |
| 4MM | 0.0013 | 0.0086 | 0.0095 | 0.0064 |  |
| 48h | | | | | |
|  | Control | Meth | Meph | MeCa | 4MM |
| Control |  | 0.0011 | 0.001 | 0.0027 | 0.002 |
| Meth | 0.0011 |  | 0.0079 | 0.0073 | 0.0074 |
| Meph | 0.001 | 0.0079 |  | 0.0069 | 0.008 |
| MeCa | 0.0027 | 0.0073 | 0.0069 |  | 0.016 |
| 4MM | 0.002 | 0.0074 | 0.008 | 0.016 |  |
| 7d | | | | | |
|  | Control | Meth | Meph | MeCa | 4MM |
| Control |  | 0.0088 | 0.0331 | 0.0089 | 0.0065 |
| Meth | 0.0088 |  | 0.0238 | 0.0076 | 0.0257 |
| Meph | 0.0331 | 0.0238 |  | 0.0619 | 0.0078 |
| MeCa | 0.0089 | 0.0076 | 0.0619 |  | 0.0092 |
| 4MM | 0.0065 | 0.0257 | 0.0078 | 0.0092 |  |
| Cell entries are p values for the indicated statistical comparisons among controls and drug treatments. | | | | | |
