# Supplementary material for: Differential effects of synthetic psychoactive cathinones and amphetamine stimulants on the gut microbiome in mice
Source: PLoS One. 2020 Jan 24;15(1):e0227774. doi: 10.1371/journal.pone.0227774 (PMC6980639; doi:10.1371/journal.pone.0227774)
Supplement: S2 Table — Cell entries are p values for the indicated statistical comparisons among controls and drug treatments. (DOCX) [file pone.0227774.s005.docx]

| **S2 Table. NPMANOVA statistical test results for Jaccard pairwise comparisons** | | | | | |
| --- | --- | --- | --- | --- | --- |
| 24h | | | | | |
|  | Control | Meth | Meph | MeCa | 4MM |
| Control |  | 0.0269 | 0.0027 | 0.0041 | 0.0052 |
| Meth | 0.0269 |  | 0.005 | 0.0108 | 0.0084 |
| Meph | 0.0027 | 0.005 |  | 0.0082 | 0.0067 |
| MeCa | 0.0041 | 0.0108 | 0.0082 |  | 0.0546 |
| 4MM | 0.0052 | 0.0084 | 0.0067 | 0.0546 |  |
| 48h | | | | | |
|  | Control | Meth | Meph | MeCa | 4MM |
| Control |  | 0.003 | 0.0057 | 0.0029 | 0.0064 |
| Meth | 0.003 |  | 0.0415 | 0.0066 | 0.0082 |
| Meph | 0.0057 | 0.0415 |  | 0.0076 | 0.0076 |
| MeCa | 0.0029 | 0.0066 | 0.0076 |  | 0.0086 |
| 4MM | 0.0064 | 0.0082 | 0.0076 | 0.0086 |  |
| 7d | | | | | |
|  | Control | Meth | Meph | MeCa | 4MM |
| Control |  | 0.0077 | 0.2694 | 0.0308 | 0.0402 |
| Meth | 0.0077 |  | 0.0153 | 0.119 | 0.0815 |
| Meph | 0.2694 | 0.0153 |  | 0.0573 | 0.0336 |
| MeCa | 0.0308 | 0.119 | 0.0573 |  | 0.0506 |
| 4MM | 0.0402 | 0.0815 | 0.0336 | 0.0506 |  |
| Cell entries are p values for the indicated statistical comparisons among controls and drug treatments. | | | | | |
